# Metabolic changes associated with two endocrine abnormalities in dogs: elevated fructosamine and low thyroxine

**DOI:** 10.1007/s11306-022-01917-4

**Published:** 2022-07-20

**Authors:** Claudia Ottka, Jenni Puurunen, Elisabeth Müller, Corinna Weber, Ruth Klein, Hannes Lohi

**Affiliations:** 1 PetBiomics Ltd, Helsinki, Finland; 2 Department of Veterinary Biosciences and Department of Medical and Clinical Genetics, University of Helsinki, Helsinki, Finland; 3 Folkhälsan Research Center, Helsinki, Finland; 4 LABOKLIN GmbH & Co KG, Bad Kissingen, Germany

**Keywords:** Endocrinology, Fructosamine, Thyroxine, Dog

## Abstract

**Introduction:**

Metabolomics studies in canine endocrine abnormalities are sparse and basic information on these abnormalities must be generated.

**Objectives:**

To characterize the metabolic changes associated with elevated fructosamine, reflecting poor glycemic control, and low thyroxine, a thyroid hormone controlling metabolism.

**Methods:**

Leftovers of clinical serum samples; 25 controls, 79 high fructosamine, and 47 low thyroxine, were analyzed using ^1^H NMR and differences were evaluated using Firth logistic regression.

**Results:**

Both high fructosamine and low thyroxine were associated with changes in concentrations of multiple metabolites, including glycoprotein acetyls and lipids.

**Conclusion:**

These findings suggest promising makers for further research and clinical validation.

**Supplementary Information:**

The online version contains supplementary material available at 10.1007/s11306-022-01917-4.

## Introduction

Endocrine disturbances are common in dogs. Since the endocrine system and metabolism are tightly intertwined, metabolism greatly affects the endocrine system, and the abnormal action of a single endocrine hormone has drastic effects on metabolism (Nelson & Couto, 2019). Metabolomics methods generate comprehensive information on metabolism and have great potential in risk assessment, diagnostics, prognostics, and treatment of endocrine diseases (Tokarz et al., 2017). For example in humans, changes in metabolite concentrations, such as branched-chain amino acids (BCAA), aromatic amino acids, and lipids have been observed years before the onset of overt type 2 diabetes mellitus, enabling preventative medicine (Ahola-Olli et al., 2019).

However, metabolomics analyses of endocrine diseases are largely unpublished in dogs (Carlos et al., 2020), and basic information on their metabolic effects is lacking. A few small-scale studies have been conducted in dogs with diabetes mellitus, demonstrating similarities to human type 1 diabetes mellitus (O’Kell et al., 2019), and one study evaluated the metabolic effects of hypo- and hyper-adrenocorticism (Imbery et al., 2022a). While the thyroid hormone thyroxine (T4) regulates metabolism, no studies have evaluated the metabolic effects of its deficiency in dogs. This study aimed to generate basic information on the metabolic changes associated with poor glycemic control, reflected by an elevated concentration of fructosamine, as well as the metabolic effects of T4 deficiency, in an animal-friendly manner using leftovers of clinical samples.

## Materials and methods

This article does not contain any studies with human or animal participants performed by any of the authors. The study was conducted utilizing leftovers of canine clinical diagnostic samples of serum submitted to a veterinary laboratory provider (LABOKLIN GmbH & Co KG, Bad Kissingen, Germany). The use of leftovers of blood samples for research purposes is expressed in the general terms and conditions of LABOKLIN GmbH & Co KG with the approval of the Government of Bavaria, Germany. Due to the samples’ origin, only limited signalment information (Online Resource S1) and no clinical data or information on sample handling before arrival at the laboratory were available.

The control group (n = 25) included samples with all standard clinical chemistry and hematology measures having normal results (Online Resource S2), and were previously used in another study (Ottka et al., 2021b). The high fructosamine group (n = 80) comprised of samples with serum fructosamine concentration above the reference limit of 374 µmol/l. The low T4 group (n = 47) included samples with serum total T4 below 1.3 µg/dl. The aliquoted sample material was stored at − 18 °C for up to 14 days before shipping on cool packs to and further storage for up to 6 months at − 80 °C prior to analysis using a validated canine nuclear magnetic resonance (NMR) spectroscopy metabolomics platform that quantifies 123 measurands (Ottka et al. 2021a). The method is described elsewhere in further detail (Ottka et al. 2021a; Soininen et al., 2009; Würtz et al., 2017).

Briefly, the method utilizes a Bruker AVANCE III HD 500 MHz NMR spectrometer with a 5 mm triple-channel (1H, 13C, 15N) z-gradient Prodigy probe head and SampleJet (Bruker Corp., Billerica, Massachusetts, USA) as the sample charger. Sample preparation includes light mixing of the sample, removal of possible precipitate, sample transfer to an NMR tube and mixing with sodium phosphate buffer using a PerkinElmer JANUS Automated Workstation with an 8-tip dispense arm with Varispan (PerkinElmer Inc., Waltham, Massachusetts, USA).

Two NMR spectra are acquired automatically: the LIPO and LMWM windows; the LIPO window comprising a conventional water-suppressed ^1^H NMR spectrum with resonances from macromolecules and lipids, whereas the LMWM window uses T2-relaxation-filtering and is used for the detection of low-molecular-weight metabolites (Soininen et al., 2009). The NMR spectra are automatically processed with background control, baseline removal, and signal alignments, and absolute metabolite concentrations are obtained with a proprietary software that bases on regression modeling, and has integrated quality control (Würtz et al., 2017).

Statistical analyses were carried out using IBM SPSS Statistics for Windows, version 27 (IBM Corp., Armonk, N.Y., USA), Microsoft Office Excel (Microsoft Corp., Redmond, WA, US) and R (R Core Team, 2020). Both case groups were evaluated against the control group separately while statistical analyses followed the same principles. First, the data were evaluated for missing observations. One sample in the high fructosamine group had over 50% missing values and was excluded (resulting n = 79). The remaining, occasional missing observations were imputed by the medians of the datasets. The data were scaled to median absolute deviation (MAD) units using median scaling. Median-based scaling methods were chosen due to the skewness and considerable variation of the data, especially in lipid measurands.

To determine associations between both studied conditions and metabolites, we created separate Firth logistic regression models for each measurand. Firth logistic regression was used to better deal with the imbalanced group sizes and the relatively low number of overall observations (Firth, 1993). The linearity of the logit was assessed, and if the linearity assumption was not met, both linear and quadratic variables were included in the final model. Bonferroni-correction was conducted based on the number of principal components explaining 95% of the variation in the dataset (Würtz et al., 2017). This was 14 in both datasets, resulting in the p value cutoff p < 0.0036. Box plots of the untransformed data were created to visualize the differences in measurand concentrations in original units compared to analysis reference intervals (Ottka et al. 2021a).

## Results and discussion

**Fig. 1:**
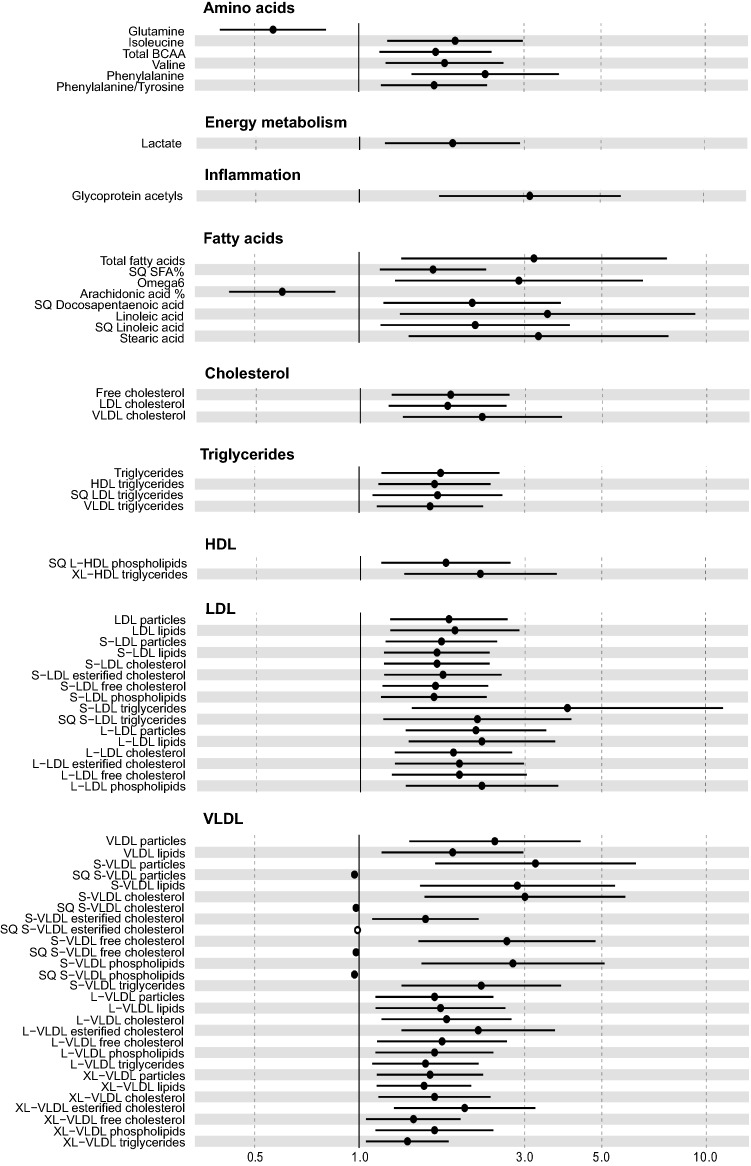
Forest plot of measurands associated (p < 0.0036) with high serum fructosamine. The odds ratio represents the strength of the measurand association to high serum fructosamine, values under 1 indicating an inverse relationship, and values over 1 indicating a direct relationship. Due to data scaling to median absolute deviation (MAD) units, a one-unit increase in the odds ratio corresponds to one MAD of the measurand. In quadratic measurands (SQ), an increased odds ratio corresponds to the squared value of the MAD scaled data. N cases = 79, n controls = 25. *CI* confidence interval, *BCAA* branched-chain amino acids, *HDL* high-density lipoprotein, *LDL* low-density lipoprotein, *VLDL* very low-density lipoprotein, *SFA* saturated fatty acids, *Omega6* omega-6 fatty acids, *L*- large, *XL*- very large, *S*- small

**Fig. 2:**
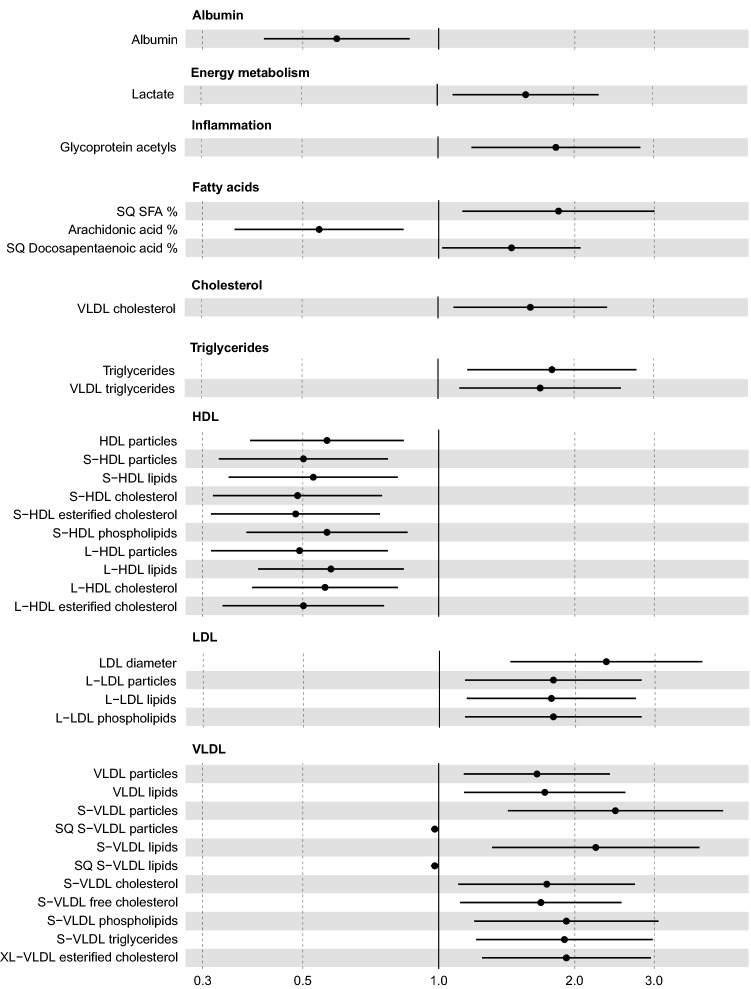
Forest plot of measurands associated (p < 0.0036) with low T4 concentration. The odds ratio represents the strength of the measurand association to low T4 concentrations, values under 1 indicating an inverse relationship, and values over 1 indicating a direct relationship. Due to data scaling to median absolute deviation (MAD) units, a one-unit increase in the odds ratio corresponds to one MAD of the measurand. In quadratic measurands (SQ), an increased odds ratio corresponds to the squared value of the MAD scaled data. N cases = 47, n controls = 25. *CI* confidence interval, *VLDL* very low-density lipoprotein, *SFA* saturated fatty acids, *HDL* high-density lipoprotein, *L*- large. *S*- small, *LDL* low-density lipoprotein, *XL*- very large

The results of the analyses are presented in Online Resource S3–S6, and in Figs. 1 and 2, and show widespread metabolic changes in both the high fructosamine and low T4 groups. One of the most interesting findings was the elevated concentration of the novel inflammatory marker GlycA in both the high fructosamine and low T4 groups. This finding holds promise for the future utility of GlycA as a general low-grade inflammatory and general disease marker in dogs. The study also shows novel information on canine lipoprotein metabolism during T4 deficiency and poor glycemic control. Furthermore, several changes in amino acid concentrations were associated with high fructosamine, and might have future value in diabetes mellitus risk evaluation and treatment.

Increased concentrations of the novel inflammatory marker GlycA increased the odds of the sample belonging to the high fructosamine group and low T4 groups, and its median concentration was outside the reference interval in the high fructosamine group. GlycA is a composite inflammatory marker elevated in multiple chronic diseases in humans, and is associated with chronic low-grade inflammation, risk of severe infection, diabetes mellitus, and even mortality (Ahola-Olli et al., 2019; Würtz et al., 2017). Until now, it has been scarcely studied in dogs, but elevated concentrations have been observed in dogs with hypo- and hyperadrenocorticism, elevated liver enzyme activities, hyperlipidemia and phenobarbital treatment (Imbery et al., 2022a, 2022b; Ottka et al. 2021a, 2021b).

Hyperlipidemia was observed in both the high fructosamine and low T4 groups, but detailed lipoprotein analysis showed different patterns of changes in lipoprotein metabolism in these groups. Elevated fructosamine was associated with widespread elevations in lipid and lipoprotein concentrations, changes in lipoproteins encompassing mainly LDL and VLDL particles, whereas significance was rarely reached for high-density lipoprotein (HDL) measures. Conversely, in the low T4 group, lipoprotein changes were focused on elevated concentrations of large LDL particles, small VLDL particles and a decrease in small and large HDL particles. While lipoprotein analyses have net previously been conducted in this detail in hypothyroid or diabetic dogs, both diseases are known to cause hyperlipidemia (Nelson & Couto, 2019; Thrall et al., 2012). Several causes are known to contribute to hyperlipidemia in diabetic dogs; impaired removal of triglycerides from VLDL and chylomicrons, increased fatty acid release to the circulation, and reduced formation of triglycerides from fatty acids in fat cells, resulting in fatty acid uptake by liver cells and formation of triglycerides packed in VLDL, and development of hypercholesterolemia due to the lack of LDL receptors and subsequently reduced LDL uptake, accompanied by increased intestinal cholesterol formation (Thrall et al., 2012).

Increasing concentrations of lactate increased the odds of the sample belonging to both the low T4 and high fructosamine groups. Elevated lactate concentrations have been previously observed in human type 2 diabetes mellitus, suggested to be caused by decreased oxidative capacity (Crawford et al., 2010). Gluconeogenesis from lactate is highly dependent on thyroid hormones (Menahan & Wieland, 1969). While resting lactate concentrations have been reported to be normal in hypothyroid humans, higher concentrations have been observed during exercise (Monzani et al., 1997). Lower albumin concentrations increased the odds of the sample belonging to the low T4 group, but its median concentration remained within reference intervals.

A unique feature of the high fructosamine group were the changes in amino acid concentrations: increasing concentrations of total BCAA and isoleucine and valine, and the aromatic amino acid phenylalanine increased, while increasing concentrations of the amino acid glutamine reduced the odds of the sample belonging to the high fructosamine group, and their median concentrations were outside analysis reference intervals. Amino acid changes are commonly observed in diabetes mellitus (Guasch-Ferre et al., 2016; O’Kell et al., 2019). High levels of phenylalanine have been associated with increased mortality risk in diabetic humans (Welsh et al., 2018). The increase in serum BCAA in diabetes mellitus may be due to disturbances in glycolysis and fatty acid oxidation, affecting muscle BCAA metabolism (Holeček, 2020). Higher concentrations of BCAA and phenylalanine have been observed already before the onset of diabetes in humans, predicting overt disease (Ahola-Olli et al., 2019; Wang et al., 2011). Furthermore, increased serum BCAA concentrations have been associated with decreased insulin sensitivity in humans (Krebs et al., 2002). Reduced glutamine levels are suggested as a possible future therapeutic target for diabetes mellitus, since glutamine supplementation has been shown to reduce hyperglycemia and to lower blood pressure in mice (Cheng et al., 2012).

The major limitation of utilizing leftovers of clinical samples is the lack of clinical information. Thus, we cannot definitively know the clinical cause of the laboratory abnormalities. Moreover, T4 concentration was not measured from all controls. Furthermore, details of the preanalytical phase before sample arrival to the laboratory are lacking. Although hyperglycemia is a hallmark of diabetes mellitus, glucose did not gain statistical significance in the Firth logistic regression analysis but showed high dispersion. This could imply that the case group samples would include treated diabetic patients, dogs with other diseases causing elevated fructosamine, or prolonged sample contact with red blood cells before serum separation, causing glucose concentrations to decline and lactate to rise (Ottka et al. 2021a). The latter could also be a reason for low outlier results for glucose. Thus, results of glucose and lactate should be interpreted with caution. However, the use of the leftover samples and comparison with well-established markers is still an excellent, animal-friendly resource for new insights and hypothesis generation (Imbery et al., 2022a, 2022b; Ottka et al., 2021b).

To summarize, we observed multiple metabolic changes associated with elevated fructosamine concentration and low T4. These results give new insights, such as detailed information on lipid metabolism, on the metabolic changes occurring during poor glycemic control or decreased thyroid hormone concentrations, indicate the need to studying the possibility of GlycA being a general marker of chronic, low-grade inflammation in dogs, and suggest further research on the roles of amino acids on diabetes mellitus development in dogs and on the possibility of amino acids being therapeutic targets of canine diabetes mellitus.

## Supplementary Information

Below is the link to the electronic supplementary material.

Supplementary file1 (XLSX 688 KB)

## Data Availability

The metabolomics and metadata reported in this paper are available at: 10.5061/dryad.tb2rbp031.
